# Circadian Analysis in Volatile Organic Compounds from *Piper gaudichaudianum* Kunth Fruits and Their Potential Role in Attracting *Carollia perspicillata* Bats

**DOI:** 10.1021/acsomega.4c08768

**Published:** 2025-01-06

**Authors:** Sirlei D. Teixeira, Thalita G. Santos, Ana Paula P. K. Hendges, Sandra B. Mikich, Gledson V. Bianconi, Francisco A. Marques, Beatriz Helena L. N. Sales Maia

**Affiliations:** † Departamento de Química, Universidade Tecnológica Federal do Paraná, Via do Conhecimento KM 01, Fraron, CP 571, 85503-390 Pato Branco, Paraná, Brazil; ‡ Departamento de Farmácia, Universidade Federal do Paraná, Av. Lothário Meissner, 632, Jardim Botânico, 80210-170 Curitiba, Paraná, Brazil; § Departamento de Química, Universidade Federal do Paraná, Av. Cel. Francisco H. dos Santos, 100, Jardim das Américas, CP 19081, 81531-980 Curitiba, Paraná, Brazil; ∥ Embrapa Florestas, Estr. Da Ribeira Br 476 Km 111, Parque Monte Castelo, CP 319, 83411-000 Colombo, Paraná, Brazil; ⊥ Instituto Federal de Educação Ciência e Tecnologia do Paraná, Câmpus Curitiba, R. João Negrão, 1285, 80230-150 Curitiba, Paraná, Brazil; # Instituto Neotropical: Pesquisa e Conservação, Rua Purus, 33, 82520-750 Curitiba, Paraná, Brazil

## Abstract

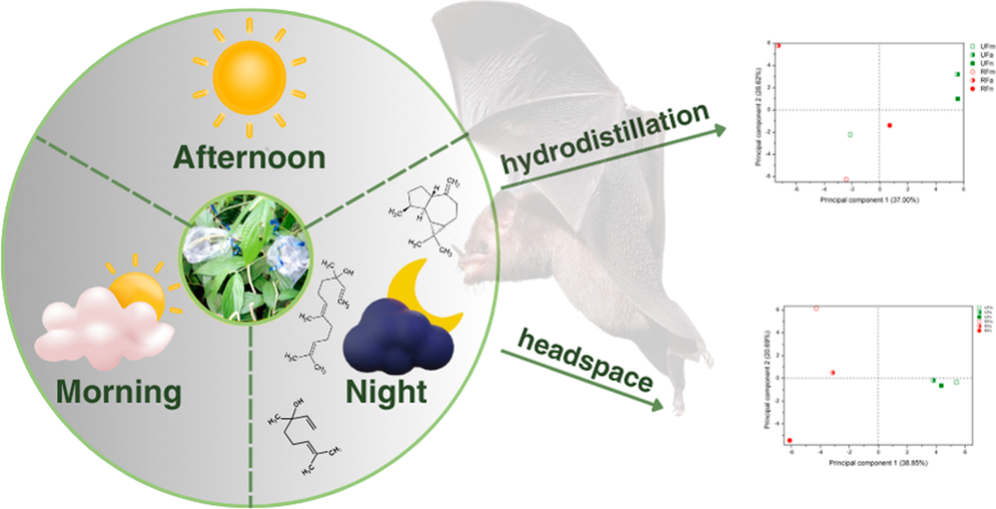

Piper fruits are one of the main dietary sources of *Carollia perspicillata*, a fruit-eating bat largely responsible for dispersing their seeds. To investigate the mechanism of this plant–animal interaction, ripe and unripe fruits of *Piper gaudichaudianum* were collected in the morning, afternoon, and night. The volatile organic compounds (VOC) were obtained through dynamic headspace (HS) and hydrodistillation (HD) and were analyzed by gas chromatography with flame ionization detector and GC–MS, resulting in the identification of ninety-five compounds. The principal component analysis of all samples revealed a distinction between the VOC obtained by the two techniques as well as differentiated the degree of ripeness of the fruits. The VOC compositions of fruits collected at night by HS and HD showed differences concerning those gathered in the morning and afternoon. The presence of linalool exclusively in the composition of ripe fruits collected at night and the highest levels of aromadendrene and (*E*)-nerolidol were also found in these samples, suggesting that these compounds may be responsible for attracting the bats and/or indicating the degree of fruit ripening. The chemical interaction between VOCs and bats appears to be more complex than simply considering the main compounds, encouraging additional tests to investigate the main compounds and their possible synergistic interactions.

## Introduction

In Brazil, fragmentation and degradation of the Atlantic Forest and associated ecosystems have reached alarming levels. For instance, in the State of Paraná, a 25% increase in the deforestation rate was recorded, comparing the periods 2016–17 with 2017–18.^1^ This alarming trend has stimulated several ecological restoration initiatives in recent years, reflecting a growing concern for the conservation of these vital ecosystems.^2^

A potential tool is the study of fruit and seed characteristics to predict dispersal processes, which can contribute to the conservation and restoration activities of tropical biodiversity. The plants produce fruits and seeds that are clearly adapted, both in terms of morphology and nutritional content, to facilitate the dispersion, since the animals develop morphological, physiological, and behavioral adaptations to find and eat the fruit, being able to disperse its seeds.^3^

Several evidence suggest that bats and small birds have an important role in the recomposition of environments by dispersing pioneer plants.^4^ In addition to the great abundance and diversity of dispersed plant species, bats can deposit seeds in specific locations for germination.^5^

The fruit-eating bat, *Carollia perspicillata*, feeds mainly on the ripe fruit of *Piper* spp.^4,6,7^ Bats are nocturnal animals, and these bats have limited vision, no color perception, high olfactory accuracy, and a poor sonar system, which suggests that the attraction to the chiropterochoric fruit may be oriented in a predominantly chemical rather than visual manner.^8−10^*Artibeus lituratus* and *C. perspicillata* respond positively to odorous stimuli reinforcing the importance of olfaction for their foraging, and these bats may be guided mainly by odor to ripe fruit.^11−14^

Our research group observed that this species could be attracted by the essential oil (EO) isolated from *Piper gaudichaudianum* (Kunth) ripe fruit inside forest remnants^11^ and remarked on the efficiency of these EOs in attracting frugivorous bats to open areas.^12^ In addition, our research group used the EO *P. gaudichaudianum* to attract frugivorous bats to regions close to areas of pasture and agriculture, suggesting that the bats attracted by the EO could have caused an increase in seed dispersal, facilitating the restoration of degraded areas.^13^

Volatile organic compounds (VOCs) are responsible for most of the aromas in plants. EOs, obtained by hydrodistillation, are mixtures of natural volatile compounds. Headspace is another method used to isolate and analyze VOCs.^15^ However, little is known about how interspecific variation in VOCs mediates mutualistic interactions with frugivores.^4^

The family Piperaceae Giseke is widely distributed around the World, and in Brazil, it is composed of 3 genera and 474 species.^16,17^ The genus *Piper* has several sympatric species that yield fruit sequentially throughout the year.^4,18^ Piper species have great ecological importance and are considered key species for forest recovery programs based on their associations with fruit-eating bats.^19^ The chemical composition of Piperaceae EOs obtained by hydrodistillation (mainly leaves and branches) has been widely studied,^20−23^ but the composition of VOCs of *P. gaudichaudianum* fruits using hydrodistillation and dynamic headspace extraction and relationship chemical constituents (submitted to principal component analysis (PCA)) with the attraction of bats is reported here for the first time.

This study aimed at investigating the composition of the VOC of ripe and unripe fruits of *P. gaudichaudianum*, collected in circadian form (morning, afternoon, and night) and obtained by two processes: hydrodistillation and dynamic headspace. As previously reported, the bat is a nocturnal animal, feeding on ripe fruits, so there is interest in knowing the VOC composition of fruits at different stages of maturation and collected at different times of the day.

With this in mind, this work aims at answering three main questions: (i) Are the VOCs obtained by dynamic headspace and hydrodistillation different? (ii) Is there an influence of the circadian cycle on the composition of VOCs in the fruits of *P. gaudichaudianum*? (iii) Do unripe and ripe fruits differ in VOC composition and concentration? Ripe fruits are expected to show a difference in the composition and concentration of VOC compared to unripe fruits, which could help bats assess fruit ripeness. The results of these observations may be useful for use in future research to attract bats, encouraging seed dispersal and subsequent forest regeneration.

## Results and Discussion

The composition of VOC of RF and UF of *P. gaudichaudianum*, obtained by hydrodistillation and dynamic headspace extraction, along with the relative abundance of their constituents, is reported in Table 1. In a general way, the VOC of *P. gaudichaudianum* fruits is mainly composed of terpenes, common substances released by plants.^24^ The yields, calculated based on the weight of fresh fruits, were determined as 0.2–0.4%, the biggest yields obtained with the hydrodistillation.

**Table 1:** VOC Obtained by Dynamic Headspace and Hydrodistillation of Unripe and Ripe Fruits of *P. gaudichaudianum* Collected in a Circadian Form (Morning, Afternoon, and Night)

			headspace						hydrodistillation					
			UFm (S1)	UFa (S2)	UFn (S3)	RFm (S4)	RFa (S5)	RFn (S6)	UFm (S7)	UFa (S8)	UFn (S9)	RFm (S10)	RFa (S11)	RFn (S12)
	compounds	RIL	RIC (%)	RIC (%)	RIC (%)	RIC (%)	RIC (%)	RIC (%)	RIC (%)	RIC (%)	RIC (%)	RIC (%)	RIC (%)	RIC (%)
	**monoterpenes hydrocarbons**		**20.87**	**51.49**	**34.31**	**32.95**	**28.05**	**40.86**		**0.51**	**0.79**	**0.89**	**5.19**	**2.29**
**1**	α-pinene	932	933 (4.66)	933 (16.96)	933 (8.81)	933 (5.22)							927 (1.09)	927 (0.29)
**2**	α-fenchene	945					938 (4.35)	938 (7.15)						
**3**	camphene	946		950 (0.21)										
**4**	β-pinene	974	980 (9.11)	981 (21.07)	981 (16.24)	979 (15.06)	985 (12.80)	985 (19.30)		979 (0.20)	979 (0.79)	979 (0.27)		979 (2.00)
**5**	myrcene	988	988 (1.71)	988 (3.66)		988 (2.80)							981 (2.82)	
**6**	δ-2-carene	1001					995 (2.34)	995 (3.32)					989 (0.10)	
**7**	α-phellandrene	1002				1010 (1.33)								
**8**	isosylvestrene	1007	1008 (0.23)				1012 (0.59)	1013 (1.06)						
**9**	δ-3-carene	1008						1015 (3.53)						
**10**	α-terpinene	1014		1010 (5.58)									1012 (0.12)	
**11**	p-cymene	1020	1024 (1.54)	1024 (0.25)										
**12**	*o*-cymene	1022			1025 (3.56)		1029 (0.59)	1029 (2.17)					1026 (0.17)	
**13**	limonene	1024	1028 (1.00)	1028 (1.30)	1029 (1.68)	1028 (2.56)	1033 (2.42)	1033 (2.87)				1030 (0.62)	1030 (0.53)	
**14**	β-phellandrene	1025			1030 (0.74)									
**15**	(*Z*)-β-ocimene	1032	1032 (0.98)	1032 (0.67)	1032 (1.17)	1032 (1.47)	1037 (1.43)						1034 (0.14)	
**16**	(*E*)-β-ocimene	1044	1042 (1.64)	1042 (1.79)	1043 (2.11)	1042 (4.51)	1047 (3.53)	1047 (1.46)		1044 (0.31)			1044 (0.22)	
	**monoterpenes oxygenated**		**0.19**		**2.73**	**11.83**	**1.70**	**0.8**		**14.85**	**17.51**			**8.38**
**17**	*o*-guaiacol	1087			1096 (2.60)					1098 (14.68)	1098 (17.26)			
**18**	linalool	1095												1098 (8.38)
**19**	(*E*)-sabinene hydrate	1098				1095 (11.83)	1101 (1.70)	1101 (0.80)						
**20**	(*Z*)-rose oxide	1106	1108 (0.19)		1108 (0.13)									
**21**	bornyl acetate	1287								1288 (0.17)	1288 (0.25)			
	**sesquiterpenes hydrocarbons**		**52.86**	**41.58**	**52.28**	**16.26**	**38.8**	**30.02**	**39.41**	**18.10**	**30.22**	**22.07**	**42.42**	**26.05**
**22**	δ-elemene	1335	1334 (0.48)	1333 (0.20)	1334 (0.41)		1339 (0.47)							
**23**	α-cubebene	1345	1349 (0.10)	1348 (0.11)	1349 (0.26)				1349 (0.12)	1349 (0.20)	1349 (0.28)			
**24**	cyclosativene	1369	1371 (0.30)	1371 (0.18)										
**25**	longicyclene	1371	1374 (0.21)	1373 (0.21)	1371 (0.50)				1371 (0.13)	1371 (0.16)			1372 (0.16)	
**26**	isoledene	1374			1374 (0.16)				1374 (0.12)				1375 (0.15)	
**27**	α-copaene	1374	1378 (3.82)	1378 (4.03)	1378 (3.93)		1383 (2.37)	1383 (2.17)	1378 (1.38)	1378 (1.24)	1378 (1.77)	1378 (1.90)		1378 (1.31)
**28**	β-patchoulene	1379				1377 (1.59)							1379 (1.98)	
**29**	β-cubebene	1387	1380 (0.71)											
**30**	β-elemene	1389	1391 (0.38)		1391 (0.22)		1396 (0.50)							
**31**	α-gurjunene	1409	1409 (0.26)	1409 (0.25)	1410 (0.67)					1409 (0.30)	1409 (0.55)		1410 (0.11)	1410 (0.59)
**32**	α-cedrene	1410		1416 (0.30)	1417 (0.36)									
**33**	(*Z*)-α-bergamotene	1411	1416 (0.30)						1415 (0.13)				1418 (0.17)	
**34**	β-caryophyllene	1417	1424 (31.11)	1425 (20.87)	1426 (21.44)	1422 (4.68)	1428 (12.07)		1423 (15.43)		1423 (8.21)	1423 (10.76)	1428 (15.74)	1423 (7.41)
**35**	β-gurjunene	1431	1433 (1.38)	1433 (0.27)	1434 (1.73)	1432 (0.86)	1438 (0.78)		1433 (0.50)	1433 (0.54)	1433 (0.68)	1433 (0.25)	1434 (0.52)	1434 (0.37)
**36**	(*E*)-α-bergamotene	1432			1435 (0.18)		1441 (0.37)							
**37**	aromadendrene	1439	1442 (1.15)	1442 (0.65)	1442 (0.99)		1447 (0.85)	1428 (15.00)		1442 (0.44)	1442 (0.57)	1442 (0.35)	1443 (0.90)	1442 (0.90)
**38**	6,9-guaiadiene	1442	1438 (1.39)	1438 (1.68)	1438 (1.04)		1444 (0.38)		1438 (0.54)				1439 (0.80)	
**39**	(*E*)-prenyl limonene	1457		1459 (2.85)	1461 (8.56)	1459 (2.80)	1465 (12.66)	1465 (8.56)	1460 (10.45)	1460 (3.54)	1460 (6.55)	1460 (2.79)	1464 (10.67)	1460 (10.80)
**40**	(*Z*)-cadina-1(6),4-diene	1461		1464 (1.95)	1465 (2.67)									
41	9-*epi*-(*E*)-caryophyllene	1464					1470 (1.09)		1464 (1.72)		1464 (0.88)	1464 (1.28)		1465 (0.31)
**42**	γ-gurjunene	1475											1467 (1.55)	
**43**	β-chamigrene	1476	1464 (3.12)						1477 (0.84)				1478 (0.93)	
**44**	γ-muurolene	1478	1476 (1.97)	1476 (1.35)	1477 (1.48)	1478 (0.97)			1479 (0.57)	1479 (1.83)	1479 (2.15)	1479 (0.61)	1480 (0.64)	1480 (0.78)
**45**	γ-himachalene	1481					1485 (1.24)							
**46**	germacrene D	1484	1479 (0.36)	1478 (0.30)	1479 (0.75)	1484 (0.60)	1491 (0.72)				1485 (0.84)			
**47**	β-selinene	1489							1494 (0.96)		1494 (0.84)			
**48**	(*Z*)-β-guaiene	1492	1485 (0.25)		1485 (1.21)					1485 (0.85)	1497 (0.65)		1485 (0.35)	
**49**	valencene	1496	1496 (0.65)	1483 (0.68)	1496 (0.54)				1496 (0.51)	1501 (1.40)			1497 (0.55)	
**50**	viridiflorene	1496												1495 (0.58)
**51**	α-selinene	1498		1493 (0.64)		1502 (2.21)	1499 (1.05)		1501 (1.92)	1494 (0.66)	1501 (1.24)	1494 (0.22)	1502 (1.16)	
**52**	bicyclogerma crene	1500	1500 (2.01)	1500 (1.10)	1501 (1.97)									
**53**	α-muurolene	1500							1503 (0.72)	1503 (0.82)	1503 (0.87)	1504 (1.17)	1505 (0.74)	1504 (0.66)
**54**	(*E*)-β-guaiene	1502					1502 (0.76)							
**55**	α-bulnesene	1509	1506 (2.28)	1506 (2.77)	1507 (1.79)		1512 (0.90)	1512 (1.16)					1508 (1.77)	
**56**	γ-cadinene	1513	1518 (0.32)	1517 (0.24)	1518 (0.49)	1517 (0.88)	1523 (1.22)	1523 (0.96)	1518 (0.55)	1518 (1.20)	1518 (1.00)	1518 (0.43)	1519 (0.57)	1519 (0.77)
**57**	7-*epi*-α-selinene	1520	1524 (0.31)											
**58**	δ-cadinene	1522		1522 (0.60)	1523 (0.93)	1522 (1.67)	1528 (1.37)	1528 (2.17)	1523 (1.20)	1523 (3.36)	1523 (2.40)	1523 (0.81)	1524 (2.02)	1523 (1.57)
**59**	(*Z*)-calamenene	1528		1526 (0.35)						1527 (0.67)	1527 (0.74)	1527 (0.58)	1528 (0.40)	
**60**	(*E*)-γ-bisabolene	1529							1524 (1.09)					
**61**	(*E*)-cadina-1,4-diene	1533								1537 (0.26)				
**62**	α-cadinene	1537								1541 (0.38)			1542 (0.11)	
**63**	α-calacorene	1544							1546 (0.26)	1546 (0.25)		1547 (0.36)	1547 (0.28)	
**64**	selina-3,7(11)-diene	1545							1541 (0.13)					
**65**	β-calacorene	1564										1568 (0.28)		
**66**	cadalene	1675							1681 (0.14)			1682 (0.28)	1682 (0.15)	
**Sesquiterpenes oxygenated**					**0.64**		**3.93**	**7.64**	**36.51**	**33.64**	**1.89**	**5.42**	**31.99**	
**67**	geranyl propanoate	1476						1471 (2.42)						
**68**	(*E*)-nerolidol	1561				1563 (0.64)		1570 (1.51)	1564 (0.90)	1566 (13.91)	1565 (11.45)			1565 (23.30)
**69**	longipinanol	1567											1561 (0.08)	
**70**	germacrene-d-4-ol	1574											1567 (2.82)	
**71**	spathulenol	1577							1582 (0.68)	1583 (2.94)	1583 (2.71)		1584 (0.25)	1583 (1.34)
**72**	caryophyllene oxide	1582							1587 (1.88)	1587 (2.76)	1587 (5.01)	1587 (1.31)	1588 (1.13)	1587 (2.66)
**73**	guaiol	1600								1591 (1.35)	1600 (1.82)		1601 (0.16)	
**74**	humulene epoxide II	1608							1615 (0.95)	1616 (4.66)	1616 (5.40)			1616 (4.06)
**75**	1,10-di-*epi*-cubenol	1618								1620 (0.44)	1620 (0.22)		1621 (0.11)	
**76**	1-*epi*-cubenol	1627								1633 (1.61)	1633 (1.01)	1634 (0.21)	1634 (0.31)	
**77**	γ-eudesmol	1630											1639 (0.12)	
**78**	α-muurolol	1644							1648 (0.63)	1640 (1.21)	1649 (1.35)	1649 (0.37)	1650 (0.44)	1650 (0.63)
**79**	cubenol	1645								1649 (2.28)				
**80**	7-*epi*-α-eudesmol	1662							1653 (0.34)	1654 (1.49)	1654 (1.20)			
**81**	(*E*)-bisabol-11-ol	1667							1663 (2.26)	1663 (3.86)	1663 (2.17)			
**82**	β-bisabolol	1674									1666 (1.30)			
	**others**		**5.02**	**3.16**	**3.13**	**4.74**	**2.53**	**10.43**	**5.09**	**1.44**	**1.55**	**6.87**	**0.15**	**0.86**
**83**	benzaldehyde	952				962 (0.43)								
**84**	naphthalene	1181	1190 (0.56)	1189 (0.18)										
**85**	*n*-dodecane	1200				1199 (0.73)	1204 (0.34)							
**86**	*n*-tridecane	1300	1301 (1.07)	1301 (1.54)	1301 (0.96)		1306 (0.41)						1301 (0.15)	
**87**	*n*-tetradecane	1400	1400 (0.24)		1401 (0.11)	1399 (1.16)	1405 (0.68)	1405 (1.46)	1400 (0.18)			1400 (0.17)		
**88**	*n*-pentadecane	1500	1502 (3.15)	1502 (1.44)	1503 (2.06)			1508 (5.64)						
**89**	butylated hydroxytoluene	1514							1507 (4.91)	1507 (1.44)	1507 (1.55)	1507 (4.95)		1508 (0.86)
**90**	*n*-hexadecane	1600				1599 (0.78)	1606 (0.46)	1606 (1.31)				1600 (0.25)		
**91**	*n*-heptadecane	1700					1708 (0.34)	1708 (0.91)						
**92**	1-octadecene	1789				1793 (0.76)						1793 (0.56)		
**93**	*n*-octadecane	1800				1799 (0.88)						1799 (0.65)		
**94**	*n*-eicosane	2000					2007 (0.30)	2007 (1.11)						
**95**	methyl-linoleate	2095										2097 (0.29)		
	**total identified**		**78.94**	**96.23**	**92.45**	**66.42**	**71.08**	**86.04**	**52.14**	**71.41**	**83.71**	**31.72**	**53.18**	**69.57**

**Figure 1:**
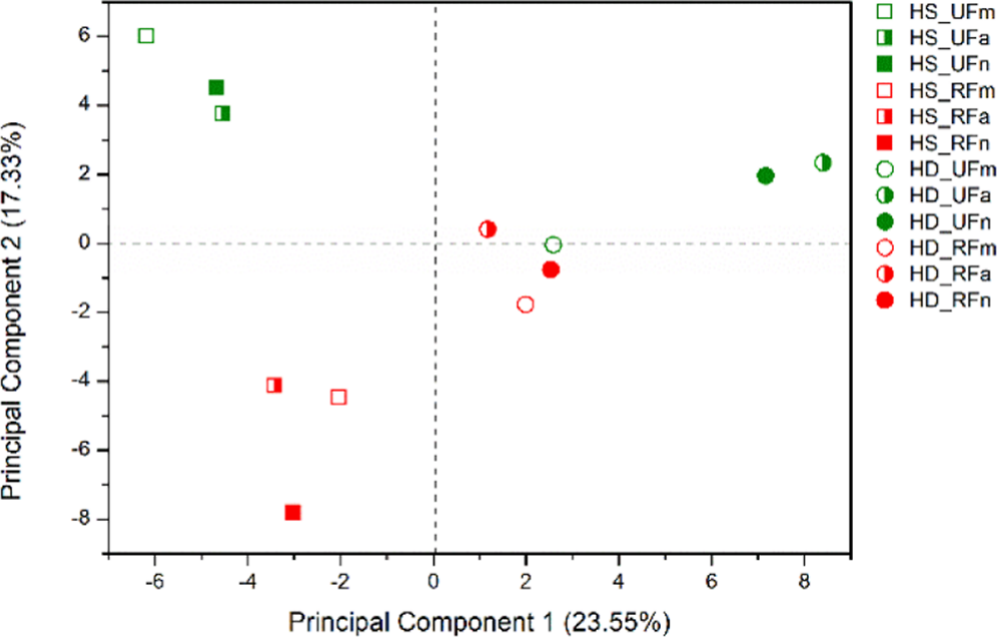
Plot of scores from the PCA of VOC from ripe and unripe fruits of *P. gaudichaudianum* obtained by dynamic headspace and hydrodistillation.

**Figure 2:**
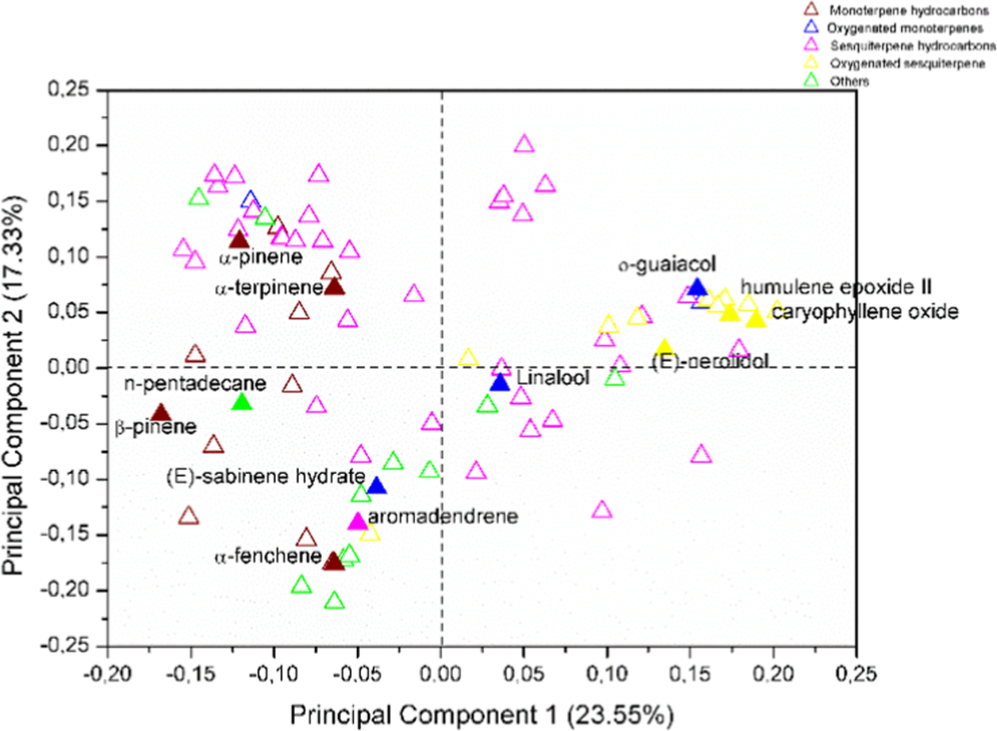
Plot of loadings from the PCA of VOC from ripe and unripe fruits of *P. gaudichaudianum* obtained by dynamic headspace and hydrodistillation. The filled symbols correspond to the compounds indicated.

In total, 95 compounds were identified by gas chromatography with flame ionization detector (GC-FID) and gas chromatography–mass spectrometry (GC–MS), accounting for 31.72–96.23% of the volatile constituents (Table 1). To verify the difference between VOCs obtained by hydrodistillation and dynamic headspace, PCA (statistical technique for linear dimensionality reduction) was performed (Figures 1 and 2). Principal component 1 (PC1) and principal component 2 (PC2) explained 40.88% of the total variance.

*Scores* showed that there is a difference in the composition of VOC extracted by HS and HD (PC1), as well as by the stage of fruit maturation (PC2). VOCs obtained by hydrodistillation are in the positive quadrant of PC1, and they have higher levels of oxygenated sesquiterpenes (1.89 to 31.99%). On the other hand, VOCs obtained by dynamic headspace are in the negative quadrant of PC1, and they have higher levels of hydrocarbon monoterpenes (20.87 to 51.49%). VOCs obtained by both extraction methods have high levels of hydrocarbon sesquiterpenes, ranging from 18.10 to 42.42% by hydrodistillation and from 16.26 to 52.86% by dynamic headspace. PC2 distinguished UF from the RF VOCs. UF VOCs are in the positive quadrant of PC2, while RF VOCs are in the negative quadrant of PC2, except for VOCs obtained by the hydrodistillation of ripe fruits collected in the afternoon, which was found in the positive quadrant of this PC.

*Loadings* have shown that *P. gaudichaudianum* VOCs obtained by hydrodistillation were mainly composed of (*E*)-nerolidol (0 to 23.3%), *o*-guaiacol (0 to 17.26%), linalool (0 to 8.38%), humulene epoxide II (0 to 5.4%), and caryophyllene oxide (1.13% to 5.01%). On the other hand, VOCs obtained by dynamic headspace were mainly composed of β-pinene (9.11 to 21.07%), α-pinene (0 to 16.96%), aromadendrene (0 to 15%), (*E*)-sabinene hydrate (0 to 11.83%), α-fenchene (0 to 7.15%), *n*-pentadecane (0 to 5.64%), and α-terpinene (0 to 5.58%). β-Caryophyllene is the major compound in both extraction methods, varying from 0 to 31.11% for dynamic headspace and 0 to 15.74% for hydrodistillation.

The VOCs present in the fruits of *P. gaudichaudianum*, obtained through dynamic headspace and hydrodistillation techniques, were different both qualitatively and quantitatively. The headspace technique effectively extracts VOCs at trace levels, providing a profile closer to reality. Additionally, it can capture subtle variations in compounds produced by the plant, enhancing our understanding of how the plant’s VOCs interact with its ecosystem. On the other hand, hydrodistillation provided a higher EO yield and, in field tests, was effective in attracting bats,^11^ despite the chemical differences in the VOCs obtained via headspace.

In a circadian study of leaves EO of *P. gaudichaudianum* collected in the Atlantic Forest of Tijuca National Park, the authors related with the set of abiotic factors, i.e., temperature, humidity, and radiation, which correlate to the day and night parameters that had more influence on the chemical composition of *P. gaudichaudianum* EOs than the variations between the dry and rainy seasons.^20^

Our study was conducted in 2020, and since then, climate change, particularly global warming, may have caused changes in the composition of EOs. Extreme temperatures affect plant physiology at various levels, potentially altering the quality and yield of the metabolites produced.^25^

**Figure 3:**
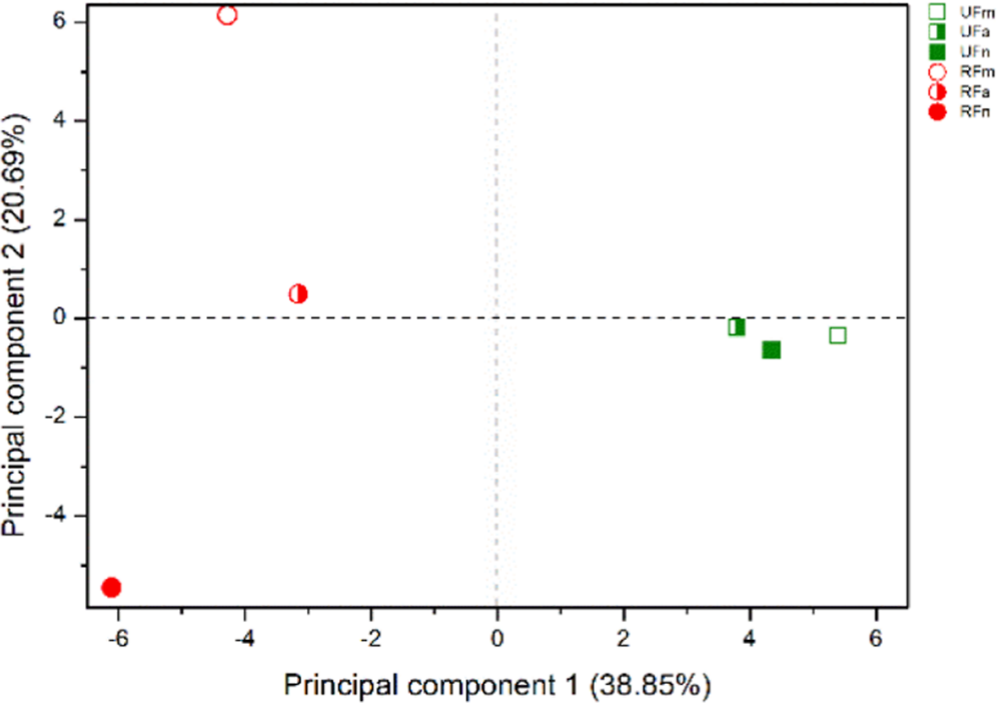
Plot of scores from the PCA of VOC from ripe and unripe fruits of *P. gaudichaudianum* obtained by dynamic headspace.

**Figure 4:**
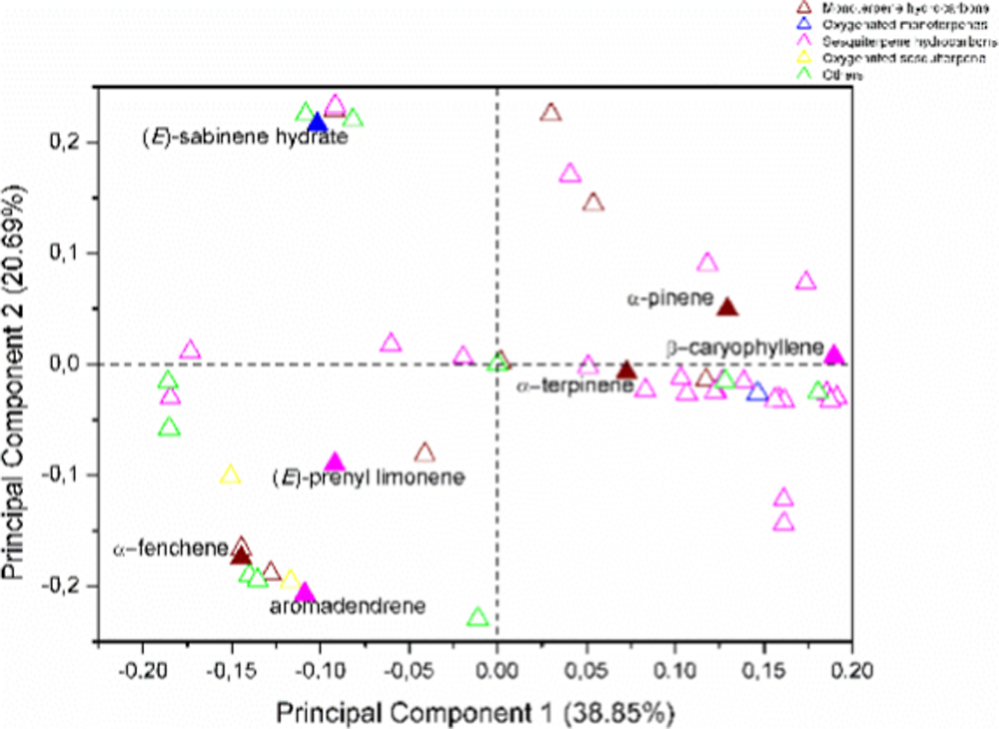
Plot of loadings from the PCA of VOC from ripe and unripe fruits of *P. gaudichaudianum* obtained by dynamic headspace. The filled symbols correspond to the compounds indicated.

**Figure 5:**
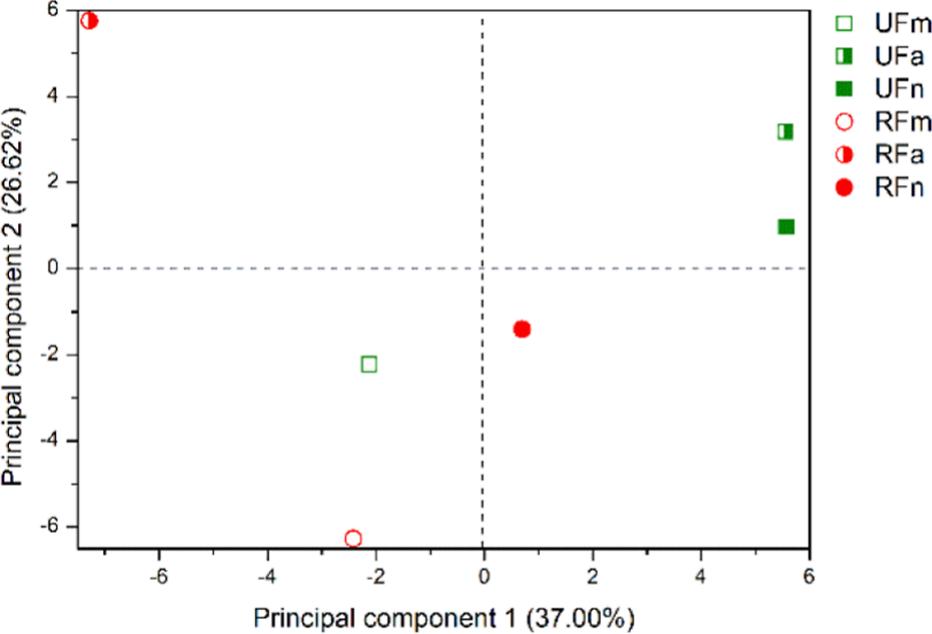
Plot of scores from the PCA of VOC from ripe and unripe fruits of *P. gaudichaudianum* obtained by hydrodistillation.

**Figure 6:**
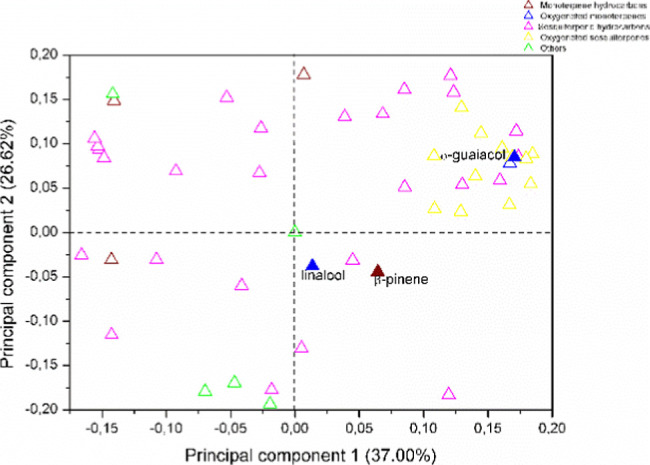
Plot of loadings from the PCA of VOC from ripe and unripe fruits of *P. gaudichaudianum* obtained by hydrodistillation. The filled symbols correspond to the compounds indicated.

Field tests run with the Ripe Fruits hydrodistillation EO confirmed the attractiveness of the EO to *C. perspicillata* since 80.7% of individuals belonging to this species were caught in the nets with mimetic fruits containing the VOC against blank ones.^11^ Through the analysis of all samples, it was possible to establish the difference between the degree of fruit maturation and the process of obtaining the VOCs. However, it is known that the bat is a nocturnal animal, and to analyze if there are some unique characteristics of nocturnality to the ripe fruit, the samples were analyzed separately, by dynamic headspace (Figures 3 and 4) and by hydrodistillation (Figures 5 and 6), since this separation was not found in total analysis.

In the PCA analysis for the VOCs obtained by dynamic headspace, Principal component 1 (PC1) and principal component 2 (PC2) explained 59.54% of the total variance. *Scores* showed a difference in the chemical composition of the UF and RF (PC1), as well as between the collection period for ripe fruits, (PC2).

UF VOCs are in the positive quadrant of PC1, and they have higher levels of sesquiterpene hydrocarbons (41.58 to 52.86%). On the other hand, RF VOCs are in the negative quadrant of PC1, with higher levels of oxygenated sesquiterpene (0 to 3.93%). An important result is that PC2 distinguished RFm and RFa from RFn VOCs, while there is not a difference for URF. *Loadings* have shown that UF VOCs were mainly composed of α-pinene (4.66 to 16.96%), α-terpinene (0 to 5.58%), and β-caryophyllene (20.87 to 31.11%), while RF VOCs were mainly composed of α-fenchene (4.35 to 7.15%), (*E*)-prenyl limonene (2.80 to 12.66%), (*E*)-sabinene hydrate (0.80 to 11.83%), and aromadendrene (0.65 to 15%).

It is important to highlight that α-fenchene and (*E*)-sabinene hydrate are present only in RF, while α-terpinene is present only in UF. Furthermore, the aromadendrene content is much higher in RF collected at night. Monoterpenes provide the initial, short-lived signal that enables bats to locate fruit trees. On the other hand, sesquiterpenes, less volatile, offer precise information about fruit ripening, allowing bats to make optimal foraging choices. These compounds also contribute to sustaining bats’ interest for longer periods, as highlighted by Parolin et al. (2019).^14^

The interaction of bats with the environment is more complex than the isolated observations. Neotropical fruit bats and omnivores (Phyllostomidae) are known to use echolocation, smell, and vision for navigation and foraging.^26^ However, the study of VOCs can contribute to the understanding of this interaction. Within this context, understanding the VOCs present in both RF and UF enhances the ecological perspective of the olfactory preferences of frugivorous bats. This could potentially prevent the extraction of VOCs from large quantities of fruits, simply by replacing them with a specific combination of monoterpenes and sesquiterpenes.^13,14,27^

In the PCA analysis for the VOC obtained by hydrodistillation, principal components 1 (PC1) and 2 (PC2) explained 63.62% of the total variance. *Scores* showed that there is a similarity between the compositions of UF VOCs collected in the afternoon and at night. These samples have higher levels of oxygenated sesquiterpene (36.51 for UFa; 33.64 for Ufn) and oxygenated monoterpenes (14.85 for UFa; 17.51 for Ufn). The composition VOC of the RF collected at night differs from the other samples, and it was observed that linalool (8.38%) was only identified in this sample. Furthermore, the highest β-pinene (2.00%) content was observed in RFn. Similarly, *o*-guaiacol was identified only in UFa (14.68%) and UFn (17.26%).

(*E*)-Nerolidol is the major compound in the samples obtained by hydrodistillation with the highest concentration found in the RF collected at night. According to Ramos et al. (2021),^20^ (*E*)-nerolidol peaked at night, increasing by up to 4-fold compared to the day. This compound is one of the main components of a “white olfactory image”, a composition that is often varied by diurnal rhythms to concentrate the effect at night to attract nocturnal visitors.

Most plants emit spikes of volatile terpenoids at noon or in the early afternoon, regulated by light or the internal circadian.^20^ This is in line with our study as the higher content of oxygenated sesquiterpenes was observed in the VOCs from UF collected in the afternoon and at night.

The chemical profile of ripe and unripe fruits differs in some specific VOCs for both techniques used; it is observed that the VOC content is higher in UF compared to RF. For fruits, it may be advantageous to provide chemical indicators that unequivocally signal the stages of ripeness. Monoterpenes and sesquiterpenes play a key role in attracting fruit bats, which use their well-developed olfactory abilities of smell to track fruit by following odor plumes carrying VOCs.^26^

The olfactory capacity of bats and their preference for ripe fruits can serve as tools in forest restoration by using fruit-derived VOCs as attractants. Understanding the chemical composition of these VOC across the circadian cycle is crucial. This approach hypothesizes that these scents can attract bats to specific areas, increasing seed dispersal and promoting natural regeneration in regions that would otherwise be rarely visited by frugivorous bats.^27^

## Conclusions

The VOCs of *P. gaudichaudianum* belong to the classes of terpenoids and hydrocarbons. PCA revealed differences between ripe and unripe fruits as well as between the VOC delivery processes by hydrodistillation and dynamic headspace. In fact, dynamic headspace sampling is usually (not always) the best technique when the olfactory impression is intended to be investigated, exactly because it is the technique that better reproduces the volatile composition perceived by the nose and olfactive system. Furthermore, the VOC composition of fruits collected at night by HS and HD differed from those collected in the morning and afternoon, which makes sense given that Neotropical bats are nocturnal. Hydrodistillation yielded a higher amount of EO, and it was effective in attracting bats in field tests, despite the chemical differences between the two extraction methods. The chemical interaction between VOCs and bats appears to be more complex than merely considering the major compounds, highlighting the need for further tests that explore the major compounds and their synergistic interactions.

## Materials and Methods

### Plant Material

Plant material was collected in a circadian form (morning, afternoon, and night) in the Parque Estadual de Vila Rica do Esprito Santo (PEVR) (23°55′00.52″S; 51°57′19.39″W, 335m), a small (354 ha) remnant of the Atlantic Forest, specifically a Semideciduous Seasonal Forest, located in Paraná state, southern Brazil. Ripe (RF) and unripe (UF) *P. gaudichaudianum* fruit were collected in January 2000, following a circadian schedule, i.e., in the morning (m), afternoon (a), and night (n), resulting in six samples: RFm, RFa, RFn, UFm, UFa, and UFn.

### Obtaining VOC

Plant samples (22 g of whole fruits) were subjected to two methods to obtain the VOC of both ripe and unripe fruits: dynamic headspace (HS) and hydrodistillation (HD). The fruits collected for hydrodistillation were stored in plastic bags and kept in a freezer at −4 °C until the VOC was obtained. The VOC was obtained in triplicate for each sample. The time to obtain the VOC of both methods was standardized in 4 h. The dynamic headspace was carried out in the field, right after harvesting the fruits. The plant material, ripe and unripe whole fruits, was placed in glass horizontal cylindrical chambers (25 cm in length × 5 cm in diameter). Fruits’ volatiles compound extraction was performed under a continuous airflow of 0.5 L min^–1^, filtered with activated carbon, to carry the volatiles released to one column (10 mm of diameter) containing 20 mg of adsorbent polymer Super Q (80–100 mesh).^28^ The desorption of the compounds retained in the adsorbent polymer was carried out using hexane solvent (1 mL), and the extracts were concentrated under nitrogen until the volume was reduced by half. Samples were kept in flasks and frozen until GC-FID and GC–MS analysis.

Hydrodistillation was performed in the laboratory with a modified Clevenger-type apparatus. The volatiles were extracted from the aqueous phase using ethyl ether and were dried with anhydrous sodium sulfate. Afterward, the filtrate (1 mL) was concentrated under nitrogen until it was reduced to half and then analyzed by GC-FID and GC–MS.

### VOC Analysis

Chemical characterization and quantification of the VOCs of *P. gaudichaudianum* fruits were carried out by gas chromatography (GC) coupled to mass spectrometry (GC–MS) and GC coupled to flame ionization detector (GC-FID), respectively, carried out immediately after obtaining the VOC. GC–MS analyses were performed on a Varian 3800 gas chromatograph (GC) equipped with a VA-5 fused silica capillary column (30 m × 0.25 mm i.d. × 0.25 μm film thickness). Temperature program: 60 to 240 °C at rate 3 °C min^–1^, column flow rate He (1 mL min^–1^), and injector temperature 250 °C. The injection volume was 1 μL, and the split was a ratio of 1:100 (for HD) or splitless (for HS). GC–MS analyses were performed on a combined GC–MS (Varian Saturn 2000) instrument, connected to an ion trap detector operating in the electron impact mode at 70 eV, a solvent delay of 3.5 min, and an interface temperature of 300 °C. The MS scan parameters included a mass range of *m*/*z* 40–350, a scan interval of 0.2 s, and a scan speed of 1666 amu s^–1^. All samples were performed in triplicate.

VOCs were quantified using an Agilent 7890 A system (GC-FID) operated at 280 °C. The column and analytical conditions were the same as those described above.

Identification of the components was based on retention indices,^29^ determined after the injection of *n*-alkane series (C_8_–C_24_) and by comparison of their MS spectra with either those stored in the NIST 2000 library and with published mass spectra.^30^ Component relative concentrations were calculated based on GC peak areas without using correction factors. Percent data shown are the mean values of three injections of each VOC sample.

### Chemical Variability

Multivariate analyses were carried out to investigate similarities between the VOC of ripe and unripe fruits of *P. gaudichaudianum* collected in the morning, afternoon, and night and extracted by dynamic headspace and hydrodistillation. PLS_toolbox 3.0 (eigenvector Research, Inc.) with MATLAB 7.0.1 software was used to perform PCA. The data were preprocessed employing autoscaling. In total, 95 chemical compounds were analyzed in 12 samples (see Table 1). First, the 12 samples were analyzed together to determine the possible differences. Next, we analyzed separately the samples obtained by HD and those obtained by HS. All graphs were generated by using Origin 8.5 software.
